# Negative Node Count Improvement Prognostic Prediction of the Seventh Edition of the TNM Classification for Gastric Cancer

**DOI:** 10.1371/journal.pone.0080082

**Published:** 2013-11-07

**Authors:** Jingyu Deng, Rupeng Zhang, Li Zhang, Yong Liu, Xishan Hao, Han Liang

**Affiliations:** Gastric Cancer Surgery Division, Tianjin Medical University Cancer Institute and Hospital, Key Laboratory of Cancer Prevention and Therapy, Tianjin, China; National Cancer Center, Japan

## Abstract

**Objective:**

To demonstrate that the seventh edition of the tumor-node-metastasis (TNM) classification for gastric cancer (GC) should be updated with the number of negative lymph nodes for the improvement of its prognostic prediction accuracy.

**Methods:**

Clinicopathological data of 769 GC patients who underwent curative gastrectomy with lymphadenectomy between 1997 and 2006 were retrospectively analyzed to demonstrate the superiority of prognostic efficiency of the seventh edition of the TNM classification, which can be improved by combining the number of negative lymph nodes.

**Results:**

With the Cox regression multivariate analysis, the seventh edition of the TNM classification, the number of negative nodes, the type of gastrectomy, and the depth of tumor invasion (T stage) were identified as independent factors for predicting the overall survival of GC patients. Furthermore, we confirmed that the T stage-N stage–number of negative lymph nodes–metastasis (TNnM) classification is the most appropriate prognostic predictor of GC patients by using case-control matched fashion and multinominal logistic regression. Finally, we were able to clarify that TNnM classification may provide more precise survival differences among the different TNM sub-stages of GC by using the measure of agreement (Kappa coefficient), the McNemar value, the Akaike information criterion, and the Bayesian Information Criterion compared with the seventh edition of the TNM classification.

**Conclusion:**

The number of negative nodes, as an important prognostic predictor of GC, can improve the prognostic prediction efficiency of the seventh edition of the TNM classification for GC, which should be recommended for conventional clinical applications.

## Introduction

Depth of primary tumor invasion and nodal metastasis are considered as the most intensive factors for predicting the prognosis of gastric cancer (GC) patients after curative surgery. The tumor-node-metastasis (TNM) classification of the Union for International Cancer Control (UICC) for GC is considered as the best classification system because of its ability to provide precise prognostic estimation and guidance for patients through appropriate therapeutic programs. Moreover, the TNM classification can distinguish the prognostic differences among various subgroups of patients by providing the anatomical extent of the primary tumor, which is currently considered as the most important prognostic predictor of GC. The TNM classification for GC has been continuously revised for several decades to improve the accuracy of its prognostic prediction. Its seventh edition shows a more meticulous prognostic sub-stage than previous editions based on the elaborate redefinitions of depth of primary tumor invasion (T stage) and nodal metastasis (N stage) [1]. More changes have been done to the N stage than the T stage in the latest edition compared with the sixth edition of the TNM classification, thereby establishing a prognostic prediction classification that mainly relies on the reform of the stage of metastatic lymph node count for GC.

Many researchers agree that the metastatic lymph node count is more appropriate in evaluating the overall survival (OS) of GC patients who underwent curative resection than the ratio between metastatic and examined lymph nodes, which is regarded as the most intensive prognostic predictor of GC as validated by previous large-scale retrospective investigations [2-5]. Recently, we adopted the N stage of the seventh edition of the TNM classification for GC in evaluating the prognosis of patients after curative surgery. The results show that the N stage of the seventh edition was significantly superior to that of the sixth edition [6]. A surgeon from Korea also reported that the seventh edition of the TNM classification for lymph node metastasis was a more reliable prognostic factor for GC than the sixth edition [7]. Although few investigators reported that the seventh edition is not superior to the other clinicopathological variables in terms of predicting the prognosis of GC [8,9], many are convinced that the seventh edition can provide a more stratified survival difference in the sub-stages of GC, which is considered to be much more reasonable compared with the sixth edition, especially between the N1- and N2-stage tumors [10-15].

Theoretically, we considered the seventh edition of the TNM classification for GC to be superior to any other clinicopathological variables. However, the tumor-ratio-metastasis (TrM) classification, which is based on the ratio between the metastatic and the examined lymph nodes, was reported to show obvious superiority to the seventh edition [16]. In addition, we previously demonstrated that the number of negative lymph nodes (NLNs) is significantly associated with the OS of GC patients after curative surgery [17]. Therefore, the aims of the present study are the following: 1) to elucidate if the seventh edition of the TNM classification is superior to the other clinicopathological variables in terms of evaluating the prognosis of GC; and 2) to identify if the prognostic prediction efficiency of the seventh edition of the TNM classification for GC can be improved by combining the number of negative nodes.

## Methods

### Patients

2326 patients with gastric cancer underwent surgical resection in the Department of Gastric Cancer Surgery, Tianjin Medical University Cancer Hospital between January 1997 and December 2006 were eligible for this study. Eligibility criteria for this study included: 1) histologically proven primary adenocarcinoma of the stomach, 2) no history of gastrectomy or other malignancy, 3) a lack of non-curative surgical factors except for distant metastasis (such as liver, lung, brain, or bone-marrow metastasis) and peritoneal dissemination, lymph node metastasis in para-aortic lymph node metastasis, 4) curative gastrectomy (subtotal or total) with lymphadenectomy performed (limited, or extended), 5) no gastroesophageal junction tumor or cardia tumor, 6) number of lymph nodes for pathological examination was no less than 15, 7) no patients died during the initial hospital stay or for 1 month after surgery. 

As a result, 1557 patients were excluded from this study. Of these excluded patients, 147 had the history of gastrectomy, 92 had other malignancy, 113 presented with hepatic metastasis intra-operation, 83 had ovarian metastasis, 204 underwent palliative gastrectomy for para-aortic node metastasis, 145 had peritoneal dissemination, 47 died of serious complications, 415 had less than 15 examined lymph nodes, and 311 identified to be gastroesophageal junction cancer or cardia cancer. Ultimately, 769 patients were included in this study.

### Surgical Treatment

All patients were operated on according to the potentially curative gastrectomy plus lymphadenectomy method. Curative resection was deﬁned as a complete lack of grossly visible tumor tissue and metastatic lymph nodes remaining after resection, with pathologically negative resection margins [18]. Primary tumors were resected en bloc with limited or extended lymphadenectomy (D1 or D2-3 according to the Japanese Gastric Cancer Association (JGCA) [19]). Limited lymphadenectomy (D1) entails the removal of the perigastric nodes only, whereas extended lymphadenectomy (D2 or D3) involves the removal of both perigastric and extragastric nodes. The choice of surgical procedure of gastrectomy (total gastrectomy or subtotal gastrectomy) was made by the attending surgeon’s preference, and based mainly on the gastric cancer treatment guidelines in Japan [20]. Surgical specimens were evaluated as recommended by the seventh edition of UICC TNM classification for gastric cancer.

### Adjuvant Therapy

135 patients (17.7%) received the adjuvant chemotherapy based on fluorouracil and leucovorin calcium after curative gastrectomy. Radiotherapy was not routinely administrated in patients routinely.

### Ethics statement

This study was approved by the Research Ethics Committee of Tianjin Medical University Cancer Institute and Hospital, and written informed consent was obtained from all patients.

### Statistics

Categorical variables were statistically compared a χ^2^ or Fisher’s exact test. Continuous data were shown as mean (s.d.) and were statistically compared using the Mann–Whitney test. To determine the most appropriate cut-off values for continuous data variables, the cut-point survival analysis [2,21] was adopted. The median OS was determined by using the Kaplan-Meier method, and log-rank test was used to determine significance. Factors that were deemed of potential importance on univariate analyses (*P*<0.05) were included in the multivariate analyses. Multivariate analysis of OS was performed by means of the Cox proportional hazards model. Hazard ratios (HR) and 95% CI were generated. The case-control matched logistic regression was used for demonstration the most intensively prognostic predictors. Linear trend χ^2^ test was used to evaluate the potential correlation among different variables. Measure of agree Kappa value and McNemar χ^2^ test were used to measure statistical consistency and homogeneity of various factors. To assess potential bias in comparing prognostic factors with different numbers of stages, the Akaike Information Criterion (AIC), and the Bayesian Information Criterion (BIC) were used. A smaller AIC or BIC value indicated a better model for predicting outcome [22,23]. Significance was defined as *P* < 0.05. All statistical analyses were performed with SPSS 18.0 software.

### Follow-up

After curative surgery, all patients were followed every 3 or 6 months for 2 year at outpatient department, every year from the third to fifth years, and then annually thereafter until the patient died. The median follow-up for the entire cohort was 51 months (range: 2-141). The follow-up of all patients who were included in this study was completed in December 2011. Ultrasonography, CT scans, chest X-ray, and endoscopy were obtained with every visit.

## Results

### Clinicopathological Outcomes

Data from 769 gastric cancer patients were analyzed. A total of 510 (66.3%) patients were male and 259 (33.7%) were female patients, with a median age of 57 years (range, 20-80 years). The median OS of all patients after curative surgery is 43.0 months, and the five year survival rates (5-YSR) is 43.8%. The patient and clinicopathological characteristics are shown in Table 1. The tumor was located in the lower third stomach in 269 (35.0%) patients, in the middle third stomach in 275 (35.8%) patients, in upper third stomach in 153 (19.9%) patients, and in more than two thirds stomach in 72 (9.3%) patients. All patients underwent the curative gastrectomy with lymphadenectomy. 577 (75.0%) patients underwent the subtotal gastrectomy, and 192 (25.0%) patients underwent the total gastrectomy. The limited lymphadenectomy was performed in 267 (34.7%) patients, and the extended lymphadenectomy was performed in 502 (65.3%) patients. Of these 769 patients, 520 (67.6%) had lymph node metastasis and 634 (82.4%) had serosal invasion. A mean of 22.81±6.95 lymph nodes per patient was dissected for histopathological examination after surgery. The mean of number of NLNs per patient was 16.35±9.40, with a range form 0 to 61. According to the seventh edition of the TNM classification for gastric cancer, 42 (5.5%) patients were Ia stage, 40 (5.2%) patients were Ib stage, 36 (4.7%) patients were IIa stage, 139 (18.1%) patients were IIb stage, 123 (16.0%) patients were IIIa stage, 128 (16.6%) patients were IIIb stage, and 261 (33.9%) were IIIc stage. 446 (58.0%) patients died when the follow-up was over. 

**Table 1:** Clinicopathologic Characteristics of the Patients Cohort.

**Gender**	
Male	510 (66.3%)
Female	259 (33.7%)
**Age at surgery**	
**Mean±SD**: 56.01 ± 11.68 years **Range**: 20 - 80 years	
< 65	554 (72.0%)
≥ 65	215 (28.0%)
**Tumor location**	
Lower third	269 (35.0%)
Middle third	275 (35.8%)
Upper third	153 (19.9%)
More than 2/3	72 (9.3%)
**Tumor size**	
**Mean±SD**: 5.40 ± 2.79 cm **Range**: 0.3 - 19.0 cm	
≤ 3.5 cm	211 (27.4%)
3.6-6.5 cm	337 (43.8%)
> 6.5 cm	221 (28.7%)
Depth of primary tumor invasion (T stage)*	
T1	45 (5.9%)
T2	42 (5.5%)
T3	48 (6.2%)
T4a	562 (73.1%)
T4b	72 (9.3%)
**Extent of lymph node metastasis**	
No	249 (32.4%)
Perigastric	374 (48.6%)
Extragastric	146 (19.0%)
**Number of examined lymph nodes**	
**Mean±SD**: 22.81 ± 6.95 **Range**: 15 - 91	
15-25	578 (75.2%)
> 25	191 (24.8%)
Number of metastatic lymph nodes (N stage)*	
**Mean±SD**: 6.46 ± 5.07 **Range**: 0 - 91	
N0	249 (32.4%)
N1	120 (15.5%)
N2	134 (17.4%)
N3a	171 (22.2%)
N3b	95 (12.4%)
**Number of negative lymph nodes (NLNs)**	
**Mean±SD**: 16.35 ± 9.40 **Range**: 0 - 61	
n1 (0-1)	23 (3.0%)
n2 (2-9)	150 (19.5%)
n3 (10-14)	137 (17.8%)
n4 (15-36)	430 (55.9%)
n5 (>36)	29 (3.8%)
**Ratio between metastatic and examined lymph nodes**	
**Mean±SD**: 27.21% ± 20.75% **Range**: 0% - 100%	
r1 (≤ 5.00%)	283 (36.8%)
r2 (5.01%-10.00%)	73 (9.5%)
r3 (10.01%-40.00%)	181 (23.5%)
r4 (40.01%-90.00%)	200 (26.0%)
r5 (> 90.00%)	32 (4.2%)
**Type of gastrectomy**	
Subtotal	577 (75.0%)
Total	192 (25.0%)
**Extent of lymphadenectomy**	
Limited	267 (34.7%)
Extended	502 (65.3%)
**Lauren’s classification of primary tumor**	
Intestinal	310 (40.3%)
Mixed	49 (6.4%)
Diffuse	410 (53.3%)
TNM Classification*	
Ia	42 (5.5%)
Ib	40 (5.2%)
IIa	36 (4.7%)
IIb	139 (18.1%)
IIIa	123 (16.0%)
IIIb	128 (16.6%)
IIIc	261 (33.9%)

SD, standard deviation.

* According to the seventh edition TNM Classification

### Univariate and Multivariate Survival Analyses

With univariate analysis, thirteen clinicopathological variables were validated to have statistically significant associations with OS of 769 patients after curative surgery. There were age at surgery (*P* = 0.002), tumor location (*P* < 0.001), tumor size (*P* < 0.001), T stage (*P* < 0.001), extent of lymph node metastasis (*P* < 0.001), number of examined lymph nodes (*P* = 0.022), N stage (*P* < 0.001), number of NLNs (*P* < 0.001), ratio between metastatic and examined lymph nodes (*P* < 0.001), type of gastrectomy (*P* < 0.001), extent of lymphadenectomy (*P* = 0.003), Lauren’s classification of primary tumor (*P* < 0.001), and the seventh edition of TNM classification for gastric cancer (*P* < 0.001). 

**Figure 1:**
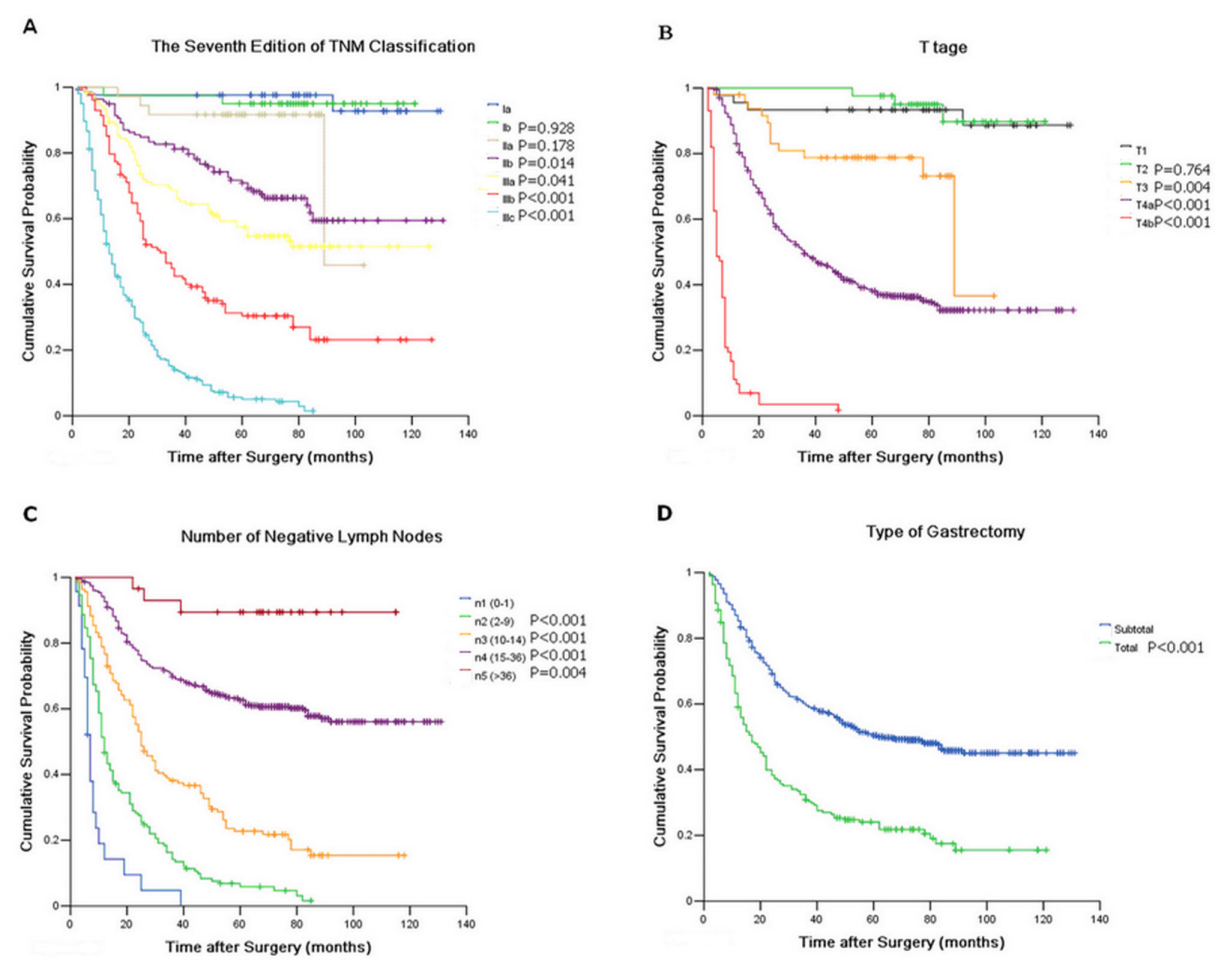
Survival curve for 769 gastric cancer patients after curative resection A) according to stage subgroup of TNM classification; B) according to stage subgroup of T stage; C) according to stage subgroup of number of negative lymph nodes; D) according to stage subgroup of type of gastrectomy.

All above thirteen variables were included in a multivariate Cox proportional hazards model (forward stepwise procedure) to adjust for the effects of covariates. With the multivariate Cox proportional hazards model (forward stepwise procedure) analysis, the seventh edition of TNM classification for GC (HR=1.797, *P* < 0.001) was identified as the independent predictor with OS of GC patients, as were T stage (HR=1.115, *P* = 0.037), number of NLNs (HR=0.752, *P* < 0.001), and type of gastrectomy (HR=1.563, *P* < 0.001) (Figure 1).

### Correlation Analysis between the Seventh Edition TNM Classification and Number of NLNs

Among the independently prognostic predictors of gastric cancer, the number of NLNs was irrelevant to the seventh edition of TNM classification for gastric cancer. However, the number of NLNs was demonstrated to have significant association with the OS of gastric cancer patients after curative surgery in our previous study[17]. Theoretically speaking, the number of NLNs should be deemed as one of the most conventionally prognostic predictor of gastric cancer for enhancement the evaluation the accuracy of lymph node dissection which may be associated with the remnants of micro-metastasis in the lymph nodes [24-27]. We do believe the number of NLNs is a essentially prognostic predictor of gastric cancer which can reflect another important aspect of primary tumor progression. With the linear trend χ^2^ test, we demonstrated the seventh edition TNM classification was significantly associated with the number of NLNs (χ^2^ value = 577.877, *P* < 0.001; likelihood ratio value = 645.455, *P* < 0.001; linear–by–linear association value = 275.360, *P* < 0.001; contingency coefficient value = 0.655, *P* < 0.001). Generally, the 5-YSR increases in the special sub-stage of the seventh edition of TNM classification as the number of NLNs increase. Therefore, we considered the number of NLNs should be a key accessorial indicator for enhancement the seventh edition of TNM classification for prediction the prognosis of gastric cancer patients after curative surgery. 

### Various Prognostic Classifications Based on the Depth of Tumor Invasion and the Status of Nodal Metastasis

**Figure 2:**
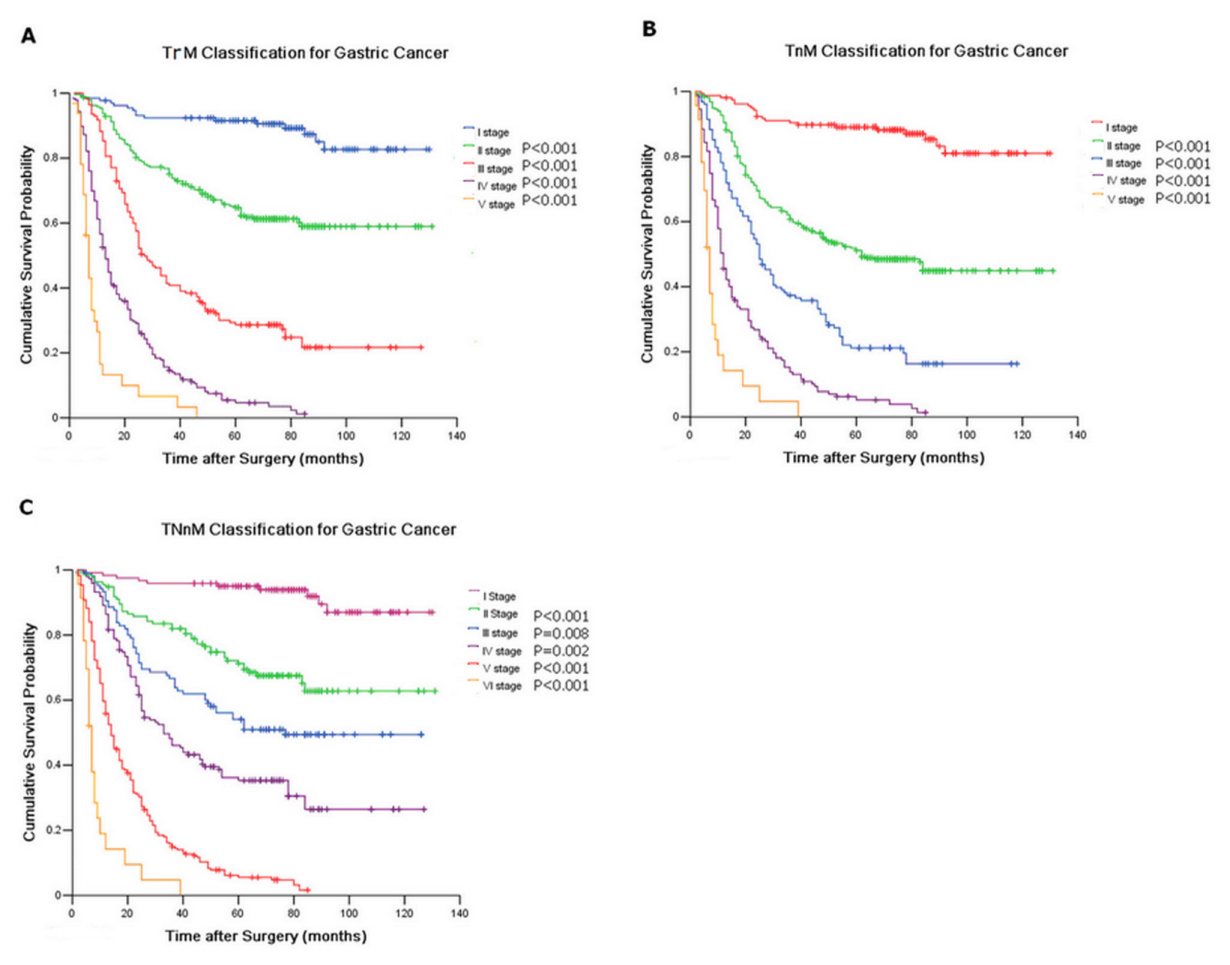
Survival curve for 769 gastric cancer patients after curative resection A) according to stage subgroup of TrM classification; B) according to stage subgroup of TnM classification; C) according to stage subgroup of TNnM classification.

We redefined various prognostic variables based on the depth of tumor invasion and status of nodal metastasis to investigate further the exact prediction for prognosis of GC. These variables include TrM classification (representing the T stage–ratio between the metastatic and the examined nodes–metastasis classification), TnM classification (representing the T stage–NLNs–metastasis classification), and the TNnM classification (representing the T stage-N stage–number of NLNs–metastasis classification). We obtained the best stage of TrM classification to evaluate the OS of GC through the appropriate cut-off value analysis by using the Kaplan-Meier method. These stages include the following: 1) I stage (including T1r1-5M0, T2r1-5M0, and T3r1-3M0), 2) II stage (including T3r4-5M0 and T4r1-2M0), 3) III stage (T4r3M0), 4) IV stage (T4r4M0), and 5) V stage (T4r5M0). Figure 2A shows the patient distribution and stage-specific survival rates, and the Kaplan-Meier plot shows the optimal discriminatory ability among the sub-stages of the TrM classification. Similarly, we obtained the best stages of TnM classification for evaluating the OS of GC through the appropriate cut-off value analysis by using the Kaplan-Meier method. These stages include the following: 1) I stage (including T1n1-5M0, T2n1-5M0, T3n1-5M0, and T4n5M0), 2) II stage (T4n4M0), 3) III stage (T4n3M0), 4) IV stage (T4n2M0), and 5) V stage (T4n1M0). Figure 2B shows the patient distribution and the stage-specific survival rates, and the Kaplan-Meier plot shows the optimal discriminatory ability among the sub-stages of TrM classification. Finally, we obtained the best stages of the TNnM classification for evaluating the OS of GC by combining the seventh edition TNM classification and the number of NLNs. These stages include the following: 1) I stage (including T1-2N0-3n1-5M0, and T3N0n1-5M0), 2) II stage (T4N0n1-5M0), 3) III stage (T3N1n4-5M0, T4N1n4-5M0), 4) IV stage (including T3N1n1-3M0, T3N2-3n1-5M0, T4N1n1-3M0, and T4N2n1-5M0), 5) V stage (T4N3n2-5M0), and 6) VI stage (T4N3n1M0). Figure 2C shows the patient distribution and stage-specific survival rates, and the Kaplan-Meier plot shows the optimal discriminatory ability among the sub-stages of TrM classification.

### Analyses of the Most Appropriately Prognostic Classification for Prediction the OS of Gastric Cancer Patients

To obtain the most appropriately prognostic classification for prediction the OS of gastric cancer patients in solid statistical method, we adopted the case-control matched logistic regression (using the forward stepwise procedure) to directly compare the different prognostic classification taking into both depth of primary tumor invasion and status of nodal metastasis. In this matched fashion, we matched 303 couple of patients (the ratio between the number of cases and the number of controls = 1:1) in accordance with variables of the type of gastrectomy (subtotal VS total), the number of examined lymph nodes (15-25 VS >25), and the extent of lymphadenectomy (limited VS extended). With the case-control matched analysis, we found the TNnM classification (HR=1.682, *P* < 0.001) was the most appropriately prognostic classification for prediction the OS of gastric cancer patients after curative surgery, rather than the seventh edition of TNM classification (*P* = 0.172), the TrM classification (*P* = 0.308), or the TnM classification (*P* = 0.001).

### Exploring the Superiorities of TNnM Classification to the Seventh Edition TNM Classification in Predicting the OS of GC Patients

**Figure 3:**
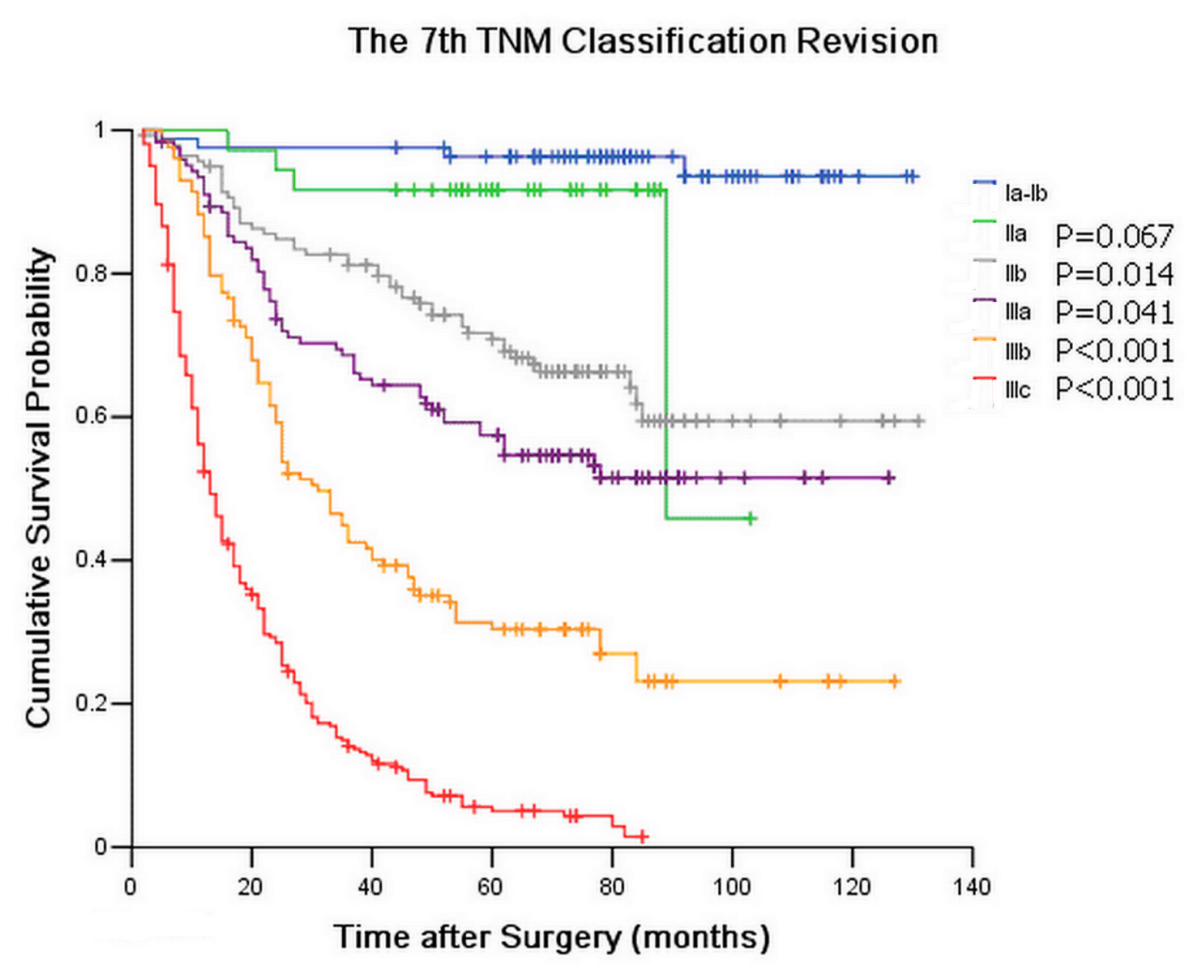
Survival curve for 769 gastric cancer patients after curative resection according to stage subgroup of TNM classification revision.

The seventh edition TNM classification for GC has been generally recognized and applied worldwide since 2009. In this study, the Kaplan-Meier plot shows the optimal discriminatory ability among the sub-stages of TNM classification except Ia and Ib (*P*=0.928). On the other hand, we also determined that the discriminatory ability among the sub-stages of TNnM classification is comprehensively distinguished. The differences in prognostic prediction between the seventh edition of the TNM classification and the TNnM classification were directly compared for convenience. We changed the sub-stages of the seventh edition TNM classification by combining Ia and Ib (Figure 3). Subsequently, both TNM and TNnM classifications had six sub-stages for further statistical analysis. Table 2 shows the patient distribution and stage-specific survival rates for different classifications. With a symmetric measure of agreement, the value of Kappa between the TNM and TNnM classifications was only 0.018, which indicates a negative statistical consistency for prognostic prediction of GC patients. In addition, McNemar χ^2^ test results show no homogeneity was observed in the prognostic prediction between the TNM and TNnM classifications (*P*<0.001).

**Table 2:** Distribution and stage specific survival rates of different classifications for prediction the prognosis of GC patients.

**Variables**	**5-YSR (%**)	**Number of patients**
**The 7th edition TNM classification**		
Ia	97.6	42
Ib	95.0	40
(Ia-Ib)	(96.3)	(82)
IIa	91.7	36
IIb	70.9	139
IIIa	57.4	123
IIIb	30.4	128
IIIc	5.1	261
**TNnM classification**		
I	95.0	121
II	71.2	134
III	54.1	106
IV	35.3	147
V	5.5	238
VI	0	23

Ultimately, we adopted the generalized multinomial logistic regression model to calculate the values of AIC and BIC to demonstrate that the TNnM classification was superior to the TNM classification in predicating the prognosis of GC patients after curative surgery. With this model, we adopted all the clinicopathological variables relative to the OS of the GC patients (TrM classification, TnM classification, and TNnM classification) as the covariates. The survival status of the patients is defined as the dependent factor in demonstrating the differences in prognostic evaluation of various variables. Based on the results, we identified that the TNnM classification had the smallest AIC and BIC values among all the covariates (AIC value=594.001 and BIC value=663.677) instead of the seventh edition of the TNM classification (AIC value=595.963 and BIC value=670.285) (Table 3).

**Table 3:** AIC and BIC values of various clinicopathological variables.

**Variables**	**AIC value**	**BIC value**
Age at surgery	595.735	665.412
Tumor location	596.834	666.510
Tumor size	595.943	665.619
T stage*	595.963	670.285
Extent of lymph node metastasis	594.230	663.906
Number of examined lymph nodes	594.486	664.162
N stage*	595.963	670.285
Number of NLNs	604.557	674.233
Ratio between metastatic and examined lymph nodes	594.270	663.947
Type of gastrectomy	600.068	669.744
Extent of lymphadenectomy	594.141	663.818
Lauren’s classification of primary tumor	597.383	667.060
TNM classification*	595.963	670.285
TrM classification	597.964	667.641
TnM classification	598.120	667.796
**TNnM classification**	**594.001**	**663.677**

* According to the seventh edition TNM Classification

## Discussion

Lymph node involvement is considered as the most important prognostic indicator of GC after curative resection regardless of the progress of primary tumor. Hitherto, most clinical investigators were in consensus that the efficiency of the number of lymph node metastasis in predicating the prognosis of GC is far better than that of the location of lymph node metastasis [28-30]. Thus, many retrospective studies have identified that the ratio between the metastatic and the examined lymph nodes was superior to the number of lymph node metastasis in terms of predicating the prognosis of GC as evidenced by the avoidance of the stage migration of malignant disease [4,31]. Recent studies have failed to demonstrate the superiority of the ratio between the metastatic and the examined lymph nodes in evaluating the prognosis of GC compared with the number of lymph node metastasis [2,3]. In theory, the ratio between the metastatic and the examined lymph nodes cannot provide complete information on both the location and the number of positive nodes of patients who had a few intraoperatively dissected lymph nodes [32]. In our previous investigation, we successfully demonstrated that the revised category of metastatic lymph node count significantly exceeded the efficiency of the ratio between the metastatic and the examined lymph nodes compared with the N stage of the sixth edition TNM classification for GC in predicating the OS of patients who had at least 15 dissected nodes by using a solid statistical method (case-control matched fashion) [31]. N stage reclassification is the major revision in the seventh edition of the TNM classification for GC, which was identified to be perfectly suitable for predicating the prognosis of GC [6,12,13]. Several supplements to the N stage were proposed to enhance the efficiency of the seventh edition TNM classification for GC in predicating the prognosis of GC patients [12,33,34]. The most optimal category for evaluating the prognosis of patients with nodal metastasis of GC still remains controversial. 

NLN count has recently been given importance for its significant association with the prognosis of patients with malignant disease [17,25,35-37]. In theory, an increasing number of examined NLNs indicates a comparatively good survival chance of patients with malignant disease after surgery with the aide of authoritative surgical curability and a sound host immune response [17,26,35]. Researchers have reported that a small proportion of GC patients with negative node metastasis examined by the conventional histological hematoxylin-eosin (HE) staining could not prevent the recurrence of GC, even after extended lymphadenectomy [38-41]. The isolated tumor cells and micrometastasis in negative lymph nodes are considered as the key factors that could lead to the adverse effect on the OS of GC patients [42-44]. Simultaneously, patients with NLN metastasis who were identified to have isolated tumor cells did not demonstrate a significantly worse prognosis than those who did not have isolated tumor cells after curative gastrectomy with extended lymphadenectomy [45]. Harrison et al [46] demonstrated that T3N0M0 GC patients who underwent extended lymphadenectomy had significantly more negative lymph nodes than those who underwent limited lymphadenectomy, thereby indicating that extended lymphadenectomy can improve the OS of T3N0M0 patients. This result is potentially associated with the elimination of micrommetastasis in NLNs. In our pervious study, we demonstrated that extended lymphadenectomy can improve the OS of GC patients with only perigastric nodal metastasis [47]. In that study, we have identified that the most important independent factor for improving the OS of GC patients with perigastric nodal metastasis after extended lymphadenectomy was the number of negative lymph nodes. In the present study, the number of NLNs was also an independent predictor of the OS of GC patients. More importantly, a significantly negative association was observed between the sub-stage of the seventh edition TNM classification and the sub-stage of the number of NLNs through the linear trend χ^2^ test. Therefore, we considered that the number of negative lymph nodes should be regarded as an indispensable predictor of the prognosis of GC after surgery, in addition to the seventh edition of TNM classification, T stage, and type of gastrectomy. Among the independent prognostic factors for GC patients after curative surgery, the T stage should be regarded as the essential part of the seventh edition of TNM classification for GC. Gastrectomy type, which is another independent prognostic factor, is a potentially subjective decision of the surgeon based on the clinical characteristics of the primary tumor in the operation. Therefore, combining the number of NLNs with the seventh edition of the TNM classification is necessary to explore the meticulous survival differences among the subgroups of GC patients after curative surgery. 

Subsequently, we designed various versions that combine the number of NLNs and the seventh edition of the TNM classification to validate the optimal classification for identifying the prognostic differences of the subgroups of GC patients after curative surgery. The TRM classification was significantly superior to the TNM classification in predicating the OS of GC more accurately; therefore, it can be used as an alternative to the TNM system [16,48]. We also considered that the ratio between the metastatic and the examined lymph nodes (including the number of NLNs) can acquire more comprehensive information such as lymph node metastasis from GC, potential micrometastasis, and host immune response than the number of metastatic lymph nodes (T stage). Unlike the TrM classification, the TNnM classification directly comprise information on both the number of metastatic nodes and the number of negative lymph nodes; thus, it can elaborately discriminate the survival differences in the subgroups of patients in the special T stage stratified by the number of negative lymph nodes. With the case-control matched test, the TNnM classification is identified as the most appropriate survival-predicating classification for GC patients after curative surgery. In this solid method analysis, we matched the type of gastrectomy to separate the surgeons’ potentially objective intervention to the bias of survival analysis. We also matched the extent of lymphadenectomy and the number of examined lymph nodes to remove the objective bias of surgeons and pathologists to the survival analysis. The results of the case-control matched test show that the TNnM classification (*P*<0.001) was the most appropriate prognostic classification for predicating the OS of GC patients after curative surgery, followed by the TnM classification (*P*=0.001). In addition, the seventh edition of the TNM classification and the TRM classification were not identified as appropriate predictors of the OS of the patients. 

We adopted the consistency χ^2^ test in exploring the symmetric level between the TNnM classification and the seventh edition of TNM classification to deduce the superiority of the TNnM classification in evaluating the postoperative prognosis of GC patients after curative surgery. Unfortunately, the Kappa value of agreement was only 0.018, which implies that the survival prediction difference of GC patients was very significant between the seventh edition TNM classification and the TNnM classification. Furthermore, the McNemar χ^2^ test results show that no homogeneity was observed in prognostic prediction between the TNM and TNnM classification. Based on the results of Figure 1A, Figure 2C, and Table 2, no overlapping survival curves or 5-YSR was observed among all the sub-stages of the TNnM classification, whereas several obviously overlapping survival curves and 5-YRS were observed among all the sub-stages of the seventh edition of the TNM classification. The most significant prognostic prediction difference between the seventh edition TNM classification and the TNnM classification for GC are as follows: 1) the survival curves of the seventh edition of the TNM classification show that no survival difference was observed among the subgroups of GC patients in Ia (97.6% of 5-YSR), Ib (95.0% of 5-YSR), and IIa (91.7% of 5-YSR) stages. Within the TNnM classification survival curves, the 5-YSR of all the patients in the I stage, 118 patients in the Ia, Ib, IIa stages, and three patients in the IIb and IIIa stages reached 95.0%. This result indicates that patients with or without subserosal invasion, with or without lymph node metastasis, can achieve optimal prognosis after curative surgery. 2) Although the number of NLNs did not demonstrate significant associations with the OS in the subgroups of the T3N0M0, T3N2M0, and T3N3M0 patients, the T3N1n4-5M0 patients had much higher 5-YSR than the T3N1n1-3M0 patients. 3) For the patients with serosal invasion, the 5-YSR decreased with increased number of metastatic lymph nodes. Moreover, a significant survival difference was observed between the T4N1n1-3M0 patients and the T4N1n4-5M0 patients. A similar phenomenon was observed in the subgroups of T4N3M0 patients with various negative lymph node counts. 4) GC patients with the worst prognosis in this study were identified in the IIIc stage in the seventh edition of the TNM classification with a 5-YSR of 5.1%, which is divided into V (238 patients with 5.5% 5-YSR) and VI (23 patients with 0% 5-YSR) stages in the TNnM classification. Among these patients, the number of NLNs was a key criterion for distinguishing the survival differences among the subgroups of patients after curative surgery (shown in Table 2). Therefore, we conclude based on the aforementioned results that the number of NLNs should not be ignored when predicating the prognosis of GC patients with more than T3N0M0 sub-stage based on the seventh edition of the TNM classification.

In conclusion, NLN count should be utilized to enhance the seventh edition of the TNM classification in evaluating the prognosis of GC after curative surgery. Admittedly, our present study is limited by the fact that it is retrospective in design and does not involve a large number of patients. However, we have initially explored and clarified that the number of NLN count is a promising indicator to be utilized when evaluating the prognosis of GC after curative surgery.

## References

[1] Sun Z, Wang ZN, Zhu Z, Xu YY, Xu Y et al. (2012) Evaluation of the seventh edition of American Joint Committee on Cancer TNM staging system for gastric cancer: results from a Chinese monoinstitutional study. Ann Surg Oncol 19: 1918-1927. doi:10.1245/s10434-011-2206-1. PubMed: 22246426. 22246426

[2] Deng JY, Liang H, Sun D, Zhan HJ, Wang XN (2008) The most appropriate category of metastatic lymph nodes to evaluate the overall survival of gastric cancer following curative resection. J Surg Oncol 98: 343-348. doi:10.1002/jso.21119. PubMed: 18668672. 18668672

[3] Bilici A, Ustaalioglu BB, Gumus M, Seker M, Yilmaz B et al. (2010) Is metastatic lymph node ratio superior to the number of metastatic lymph nodes to assess outcome and survival of gastric cancer? Onkologie 33: 101-105. doi:10.1159/000277927. PubMed: 20215800. 20215800

[4] Marchet A, Mocellin S, Ambrosi A, Morgagni P, Garcea D, et al (2007) The ratio between metastatic and examined lymph nodes (N ratio) is an independent prognostic factor in gastric cancer regardless of the type of lymphadenectomy: results from an Italian multicentric study in 1853 patients. Ann Surg 245: 543-552. doi:10.1097/01.sla.0000250423.43436.e1. PubMed: 17414602. 17414602 PMC1877031

[5] Lee SY, Hwang I, Park YS, Gardner J, Ro JY (2010) Metastatic lymph node ratio in advanced gastric carcinoma: a better prognostic factor than number of metastatic lymph nodes? Int J Oncol 36: 1461-1467. PubMed: 20428770. 20428770 10.3892/ijo_00000632

[6] Deng JY, Liang H, Sun D, Wang D, Pan Y (2010) Suitability of 7th UICC N stage for predicting the overall survival of gastric cancer patients after curative resection in China. A Single Clinical Center Report of New N Stage. Ann Surg Oncol 17: 1259-1266. doi:10.1245/s10434-010-0939-x. PubMed: 20217252. 20217252

[7] Chae S, Lee A, Lee JH (2011) The effectiveness of the new (7th) UICC N classification in the prognosis evaluation of gastric cancer patients: a comparative study between the 5th/6th and 7th UICC N classification. Gastric Cancer 14: 166-171. doi:10.1007/s10120-011-0024-6. PubMed: 21360132. 21360132

[8] Warneke VS, Behrens HM, Hartmann JT, Held H, Becker T et al. (2011) Cohort study based on the seventh edition of the TNM classification for gastric cancer: proposal of a new staging system. J Clin Oncol 29: 2364-2371. doi:10.1200/JCO.2010.34.4358. PubMed: 21537040. 21537040

[9] Yoon HM, Ryu KW, Nam BH, Cho SJ, Park SR et al. (2012) Is the new seventh AJCC/UICC staging system appropriate for patients with gastric cancer? J Am Coll Surg 214: 88-96. doi:10.1016/j.jamcollsurg.2011.09.018. PubMed: 22036661. 22036661

[10] Ahn HS, Lee HJ, Hahn S, Kim WH, Lee KU et al. (2010) Evaluation of the Seventh American Joint Committee on Cancer/International Union Against Cancer Classification of gastric adenocarcinoma in comparison with the sixth classification. Cancer 116: 5592-5598. doi:10.1002/cncr.25550. PubMed: 20737569. 20737569

[11] Qiu MZ, Wang ZQ, Zhang DS, Liu Q, Luo HY et al. (2011) Comparison of 6th and 7th AJCC TNM staging classification for carcinoma of the stomach in China. Ann Surg Oncol 18: 1869-1876. doi:10.1245/s10434-010-1542-x. PubMed: 21246404. 21246404

[12] Marrelli D, Morgagni P, de Manzoni G, Coniglio A, Marchet A, et al (2012) Prognostic value of the 7th AJCC/UICC TNM classification of noncardia gastric cancer: analysis of a large series from specialized Western centers. Ann Surg 255: 486-491. doi:10.1097/SLA.0b013e3182389b1a. PubMed: 22167003. 22167003

[13] Wang W, Sun XW, Li CF, Lv L, Li YF et al. (2011) Comparison of the 6th and 7th editions of the UICC TNM staging system for gastric cancer: results of a Chinese single-institution study of 1,503 patients. Ann Surg Oncol 18: 1060-1067. doi:10.1245/s10434-010-1424-2. PubMed: 21107742. 21107742 PMC3052465

[14] Fang WL, Huang KH, Chen JH, Lo SS, Hsieh MC et al. (2011) Comparison of the survival difference between AJCC 6th and 7th editions for gastric cancer patients. World J Surg 35: 2723-2729. doi:10.1007/s00268-011-1275-4. PubMed: 21918892. 21918892

[15] Kikuchi S, Futawatari N, Sakuramoto S, Katada N, Yamashita K et al. (2011) Comparison of staging between the old (6th edition) and new (7th edition) TNM classifications in advanced gastric cancer. Anticancer Res 31: 2361-2365. 21737665

[16] Wang W, Xu DZ, Li YF, Guan YX, Sun XW et al. (2011) Tumor-ratio-metastasis staging system as an alternative to the 7th edition UICC TNM system in gastric cancer after D2 resection--results of a single-institution study of 1343 Chinese patients. Ann Oncol 22: 2049-2056. doi:10.1093/annonc/mdq716. PubMed: 21310759. 21310759

[17] Deng JY, Liang H, Wang DC, Sun D, Ding X et al. (2010) Enhancement the prediction of postoperative survival in gastric cancer by combining the negative lymph node count with ratio between positive and examined lymph nodes. Ann Surg Oncol 17: 1043-1051. doi:10.1245/s10434-009-0863-0. PubMed: 20039218. 20039218

[18] Hermanek P, Wittekind C (1994) Residual tumor (R) classiﬁcation and prognosis. Semin Surg Oncol 10: 12–20. doi:10.1002/ssu.2980100105. PubMed: 8115781. 8115781

[19] Jaehne J, Meyer HJ, Maschek H, Geerlings H, Burns E et al. (1992) Lymphadenectomy in gastric adenocarcinoma: a prospective and prognostic study. Arch Surg 127: 290–294. doi:10.1001/archsurg.1992.01420030052010. PubMed: 1372495. 1372495

[20] Nakajima T (2002) Gastric cancer treatment guidelines in Japan. Gastric Cancer; 5: 1–5. doi:10.1007/s10120-002-0206-3. PubMed: 12021853. 12021853

[21] Smith DD, Schwarz RR, Schwarz RE (2005) Impact of total lymph node count on staging and survival after gastrectomy for gastric cancer: data from a large US-Population Database. J Clin Oncol 23: 7114-7124. doi:10.1200/JCO.2005.14.621. PubMed: 16192595. 16192595

[22] Nitsche U, Maak M, Schuster T, Künzli B, Langer R et al. (2011) Prediction of prognosis is not improved by the seventh and latest edition of the TNM classification for colorectal cancer in a single-center collective. Ann Surg 254: 793-800. doi:10.1097/SLA.0b013e3182369101. PubMed: 22042471. 22042471

[23] Cho YK, Chung JW, Kim JK, Ahn YS, Kim MY et al. (2008) Comparison of 7 staging systems for patients with hepatocellular carcinoma undergoing transarterial chemoembolization. Cancer 112: 352-361. doi:10.1002/cncr.23185. PubMed: 18008352. 18008352

[24] Schwarz RE, Smith DD (2007) Clinical impact of lymphadenectomy extent in respectable gastric cancer of advanced stage. Ann Surg Oncol; 14: 317-328. doi:10.1245/s10434-006-9218-2. PubMed: 17094022. 17094022

[25] Johnson PM, Porter GA, Ricciardi R, Baxter NN (2006) Increasing negative lymph node count is independently associated with improved long-term survival in stage IIIB and IIIC colon cancer. J Clin Oncol 24: 3570-3575. doi:10.1200/JCO.2006.06.8866. PubMed: 16877723. 16877723

[26] Kim JJ, Song KY, Hur H, Hur JI, Park SM et al. (2009) Lymph node micrometastasis in node negative early gastric cancer. Eur J Surg Oncol 35: 409-414. doi:10.1016/j.ejso.2008.05.004. PubMed: 18573635. 18573635

[27] Saito H, Osaki T, Murakami D, Sakamoto T, Kanaji S et al. (2007) Recurrence in early gastric cancer--presence of micrometastasis in lymph node of node negative early gastric cancer patient with recurrence. Hepatogastroenterology 54: 620-624. PubMed: 17523336. 17523336

[28] Wu CW, Hsieh MC, Lo SS, Shen KH, Lui WY et al. (2001) Comparison of the UICC/AJCC 1992 and 1997 pN categories for gastric cancer patients after surgery. Hepato Gastroenterol; 48: 279-284. PubMed: 11268985. 11268985

[29] Katai H, Yoshimura K, Maruyama K, Sasako M, Sano T (2000) Evaluation of the New International Union Against Cancer TNM staging for gastric carcinoma. Cancer 88: 1796-1800. doi:10.1002/(SICI)1097-0142(20000415)88:8. PubMed: 10760754. 10760754

[30] Adachi Y, Kamakura T, Mori M, Baba H, Maehara Y (1994) Prognostic significance of the number of positive lymph nodes in gastric carcinoma. Br J Surg; 81: 414–416. doi:10.1002/bjs.1800810331. PubMed: 8173916. 8173916

[31] Maduekwe UN, Lauwers GY, Fernandez-Del-Castillo C, Berger DL, Ferguson CM (2010) New metastatic lymph node ratio system reduces stage migration in patients undergoing D1 lymphadenectomy for gastric adenocarcinoma. Ann Surg Oncol; 17: 1267-1277. doi:10.1245/s10434-010-0914-6. PubMed: 20099040. 20099040 PMC3785005

[32] Kulig J, Sierzega M, Kolodziejczyk P, Popiela T, Polish Gastric Cancer Study Group (2009) Ratio of metastatic to resected lymph nodes for prediction of survival in patients with inadequately staged gastric cancer. Br J Surg 96: 910-918. doi:10.1002/bjs.6653. PubMed: 19591164. 19591164

[33] Gobbi PG, Villano L, Pozzoli D, Bergonzi M, Vanoli A et al. (2011) Improving the AJCC/TNM classification for use in early gastric cancer. J Gastrointest Surg 15: 935-941. doi:10.1007/s11605-011-1522-x. PubMed: 21484483. 21484483

[34] Wang J, Dang P, Raut CP, Pandalai PK, Maduekwe UN et al. (2012) Comparison of a lymph node ratio-based staging system with the 7th AJCC system for gastric cancer: analysis of 18,043 patients from the SEER database. Ann Surg 255: 478-485. doi:10.1097/SLA.0b013e31824857e2. PubMed: 22330040. 22330040

[35] Ogino S, Nosho K, Irahara N, Shima K, Baba Y et al. (2010) Negative lymph node count is associated with survival of colorectal cancer patients, independent of tumoral molecular alterations and lymphocytic reaction. Am J Gastroenterol 105: 420-433. doi:10.1038/ajg.2009.578. PubMed: 19809407. 19809407 PMC2878181

[36] Ampil FL, Caldito G, Ghali GE, Baluna RG (2009) Does the negative node count affect disease-free survival in early-stage oral cavity cancer? J Oral Maxillofac Surg 67: 2473-2475. doi:10.1016/j.joms.2009.04.126. PubMed: 19837320. 19837320

[37] Huang CM, Lin JX, Zheng CH, Li P, Xie JW et al. (2011) Effect of negative lymph node count on survival for gastric cancer after curative distal gastrectomy. Eur J Surg Oncol 37: 481-487. doi:10.1016/j.ejso.2011.01.012. PubMed: 21371852. 21371852

[38] Arigami T, Uenosono Y, Yanagita S, Nakajo A, Ishigami S et al. (2013) Clinical significance of lymph node micrometastasis in gastric cancer. Ann Surg Oncol 20: 515-521. doi:10.1245/s10434-012-2355-x. PubMed: 22546997. 22546997

[39] Yano K, Nimura H, Mitsumori N, Takahashi N, Kashiwagi H et al. (2012) The efficiency of micrometastasis by sentinel node navigation surgery using indocyanine green and infrared ray laparoscopy system for gastric cancer. Gastric Cancer 15: 287-291. doi:10.1007/s10120-011-0105-6. PubMed: 22041868. 22041868

[40] Cao L, Hu X, Zhang Y, Huang G (2011) Adverse prognosis of clustered-cell versus single-cell micrometastases in pN0 early gastric cancer. J Surg Oncol 103: 53-56. doi:10.1002/jso.21755. PubMed: 21031429. 21031429

[41] Ishida K, Katsuyama T, Sugiyama A, Kawasaki S (1997) Immunohistochemical evaluation of lymph node micrometastases from gastric carcinomas. Cancer 79: 1069-1076. doi:10.1002/(SICI)1097-0142(19970315)79:6. PubMed: 9070482. 9070482

[42] Yonemura Y, Endo Y, Hayashi I, Kawamura T, Yun HY et al. (2007) Proliferative activity of micrometastases in the lymph nodes of patients with gastric cancer. Br J Surg 94: 731-736. doi:10.1002/bjs.5604. PubMed: 17377930. 17377930

[43] Yanagita S, Natsugoe S, Uenosono Y, Kozono T, Ehi K et al. (2008) Sentinel node micrometastases have high proliferative potential in gastric cancer. J Surg Res 145: 238-243. doi:10.1016/j.jss.2007.04.037. PubMed: 17603078. 17603078

[44] Kim JH, Park JM, Jung CW, Park SS, Kim SJ et al. (2008) The significances of lymph node micrometastasis and its correlation with E-cadherin expression in pT1-T3N0 gastric adenocarcinoma. J Surg Oncol 97: 125-130. doi:10.1002/jso.20937. PubMed: 18095267. 18095267

[45] Fukagawa T, Sasako M, Shimoda T, Sano T, Katai H et al. (2009) The prognostic impact of isolated tumor cells in lymph nodes of T2N0 gastric cancer: comparison of American and Japanese gastric cancer patients. Ann Surg Oncol 16: 609-613. doi:10.1245/s10434-008-0290-7. PubMed: 19137375. 19137375

[46] Harrison LE, Karpeh MS, Brennan MF (1998) Extended lymphadenectomy is associated with a survival benefit for node-negative gastric cancer. J Gastrointest Surg 2: 126-131. doi:10.1016/S1091-255X(98)80002-4. PubMed: 9834407. 9834407

[47] Deng JY, Liang H, Sun D, Pan Y, Liu Y et al. (2011) Extended lymphadenectomy improvement of overall survival of gastric cancer patients with perigastric node metastasis. Langenbecks Arch Surg 396: 615-623. doi:10.1007/s00423-011-0753-3. PubMed: 21380618. 21380618

[48] Persiani R, Rausei S, Antonacci V, Biondi A, Casella F et al. (2009) Metastatic lymph node ratio: a new staging system for gastric cancer. World J Surg 33: 2106-2111. doi:10.1007/s00268-009-0157-5. PubMed: 19636611. 19636611

